# Parent tRNA Modification Status Determines the Induction of Functional tRNA-Derived RNA by Respiratory Syncytial Virus Infection

**DOI:** 10.3390/v15010057

**Published:** 2022-12-24

**Authors:** Eun-Jin Choi, Wenzhe Wu, Ke Zhang, Xiaohong Yuan, Junfang Deng, Deena Ismail, Darby L. Buck, Kerrie S. Thomason, Roberto P. Garofalo, Shenglong Zhang, Xiaoyong Bao

**Affiliations:** 1 Department of Pediatrics, University of Texas Medical Branch, Galveston, TX 77555, USA; 2 Department of Biological and Chemical Science, New York Institute of Technology, New York, NY 10023, USA; 3 Institute of Translational Science, University of Texas Medical Branch, Galveston, TX 77555, USA; 4 Institute for Human Infections Immunity, University of Texas Medical Branch, Galveston, TX 77555, USA

**Keywords:** tRF, tRNA modification, RSV replication

## Abstract

tRNA-derived RNA fragments (tRFs) are a recently discovered family of small noncoding RNAs (sncRNAs). We previously reported that respiratory syncytial virus (RSV) infection induces functional tRFs, which are derived from a limited subset of parent tRNAs, in airway epithelial cells. Such induction is also observed in nasopharyngeal wash samples from RSV patients and correlates to RSV genome copies, suggesting a clinical significance of tRFs in RSV infection. This work also investigates whether the modification of parent tRNAs is changed by RSV to induce tRFs, using one of the most inducible tRFs as a model. We discovered that RSV infection changed the methylation modification of adenine at position 57 in tRNA glutamic acid, with a codon of CTC (tRNA-GluCTC), and the change is essential for its cleavage. AlkB homolog 1, a previously reported tRNA demethylase, appears to remove methyladenine from tRNA-GluCTC, prompting the subsequent production of tRFs from the 5′-end of tRNA-GluCTC, a regulator of RSV replication. This study demonstrates for the first time the importance of post-transcriptional modification of tRNAs in tRF biogenesis following RSV infection, providing critical insights for antiviral strategy development.

## 1. Introduction

Respiratory syncytial virus (RSV) is a negative-sense single-stranded non-segmented RNA virus. As an orthopneumovirus, it belongs to the Pneumoviridae family and is the most significant cause of lower respiratory tract infections (LRTIs) in the pediatric population [1,2]. Other than palivizumab, which prevents RSV infection in high-risk infants, there is no vaccine or virus-specific pharmaceutic treatment for RSV [3], demonstrating the need to expand our current knowledge on viral replication mechanisms.

Noncoding RNAs (ncRNAs) comprise most of the human genome (as compared with protein-coding RNAs) [4,5], and their functions in physiological and pathological settings are increasingly recognized. tRNA-derived RNA fragments (tRFs) are a recently discovered category of ncRNAs whose importance in viral infectious diseases is becoming more evident [6,7,8,9,10]. We have recently shown that RSV infection induces a significant increase in tRFs. These induced tRFs are not byproducts of tRNA degradation but functional molecules because (1) the induction is virus-specific, (2) only a selective subset of parent tRNAs are cleaved in a tRNA structure-dependent manner, and (3) some are functionally important in viral replication and regulate genes using mechanisms different from miRNAs [8,11]. We also found that ribonuclease angiogenin (ANG) controls most cleaved tRNAs in RSV while ElaC ribonuclease Z 2 (ELAC2) is essential for the cleavage of tRNA-GlnCTG [8,11,12]. However, the cause of tRNAs being prone to cleavage is not fully understood.

tRF derived from the 5′-end of tRNA-encoding GluCTC (tRF5-GluCTC) is heavily impacted by RSV. It has also shown its importance in other diseases, including cancer and neurodegenerative disease [13,14,15], and is also inducible by SARS-CoV-2 infection. Therefore, we used tRF5-GluCTC as a start to study the biogenesis mechanism of tRF5s. Furthermore, since tRNAs are highly modified molecules, and the modification status of RNAs plays a role in RNA stability [16,17,18], we investigated whether RSV infection ignites the cleavage of parent tRNA-GluCTC by changing its modification status. Herein, our cutting-edge de novo mass spectrometric (MS)-based sequencing methods, known as MS-Seq, enabled us to directly detect a decrease in adenine (A) methylation at position 57 in tRNA-GluCTC. AlkB homolog 1 (ALKBH1), a reported tRNA demethylase with a demethylation preference for methyladenine present on a T loop structure of tRNAs [19], played a critical role in tRNA-GluCTC cleavage following the RSV infection, demonstrating that ALKBH1-mediated A demethylation is critical in the induction of tRF5-GluCTC. We also found a novel modification change on two pseudoruidine residues by RSV, awaiting the identification of associated modification enzymes. Since tRF5-GluCTC promotes RSV replication [8,13], the importance of tRNA modification to tRF biogenesis provides new insights for viral replication control.

## 2. Materials and Methods

### 2.1. Nasopharyngeal Secretions (NPS) from Patients

Samples of NPS were collected by gentle suction of the nasopharynx from hospitalized children who were enrolled in a study on the pathogenesis of RSV bronchiolitis under protocol #03-117 (approved by the Institutional Review Board of UTMB). The Luminex NxTAG™ Respiratory Pathogen Panel (RPP, Luminex Molecular Diagnostics, Austin, TX, USA) was used to detect RSV and other viruses in NPS samples. Only RSV-positive NPS (*n* = 9) were used as positive samples and pathogen-negative NPS (*n* = 8) as controls. The information on the patient’s gender and age is shown in Table 1.

### 2.2. Cells and Virus

A549 (human alveolar type II-like epithelial) and HEp-2 (human epithelial type 2) were from the ATCC (Manassas, VA) and maintained as previously described [20]. RSV long strain was propagated in HEp-2 cells at 37 °C, and the virus purification was done by sucrose gradient as described previously [20,21]. RSV titer was determined by immunostaining in HEp-2 cells using polyclonal biotin-conjugated goat anti-RSV antibody (Bio-Rad, Hercules, CA, USA) and streptavidin peroxidase polymer, as previously described [20,21].

### 2.3. RNA Preparation

To prepare RNAs from patient samples, RNAs from 300 µL of NPS, after adding cel-miR-39 as reference controls for sample preparation, were harvested using *mir*Vana^TM^ Protein and RNA Isolation System from Invitrogen (Waltham, MA, USA, Cat# AM1556), following the manufacturer’s protocol. To harvest RNAs from cell cultures, we used Trizol from Invitrogen (Cat# 15596-026) to extract RNAs as we previously described in [20,22,23].

### 2.4. qRT-PCR

Trizol-extracted RNAs or RNAs from patient NPS were used to examine viral replication by qRT-PCR, as previously described for RSV or its family member, human metapeumovirus [20,22,23]. In brief, to quantify viral genomic copies of RSV, we used primers to assess the genomic part between the N and P regions. First-strand cDNA was transcribed with a P-specific primer: 5′-CGTCTCAGCCAATCCCTGGTGATTATGAGTAATTAAAAAATGGGACAAG-3′. The underlined letters indicate the chloramphenicol resistance (Cmr) tag sequence, which was designed as part of the assay to prevent self-priming exhibited by viral RNA [24]. QPCRs were performed using the forward primer 5′-CGTCTCAGCCAATCCCTGG-3′ and the reverse primer 5′-GCTTCATTACCCATGAAAAGAATATC-3′.

To quantify NPS tRF expression, we used modified qRT-PCR that can differentiate tRF signals from its parent tRNA, as previously described in [9]. In brief, RNAs were treated with T4 PNK and then ligated to a 3′-RNA linker using T4 RNA ligase from Thermo Fisher Scientific (Waltham, MA, USA). The product was used as a template for RT with a linker-specific reverse primer using TaqMan Reverse Transcription Reagents from Thermo Fisher Scientific. The cDNA was subjected to SYBR Green qPCR using iTaqTM Universal SYBR Green Supermix kit from Bio-Rad (Hercules, CA, USA) and primers specific to the 5′-end of tRFs and RNA linker. U6 and externally added cel-miR-39 were used for normalization.

### 2.5. tRNA-GluCTC Sample Preparation

Ten T-150 flasks of confluent A549 cells were infected with RSV at a multiplicity of infection (MOI) of 1. An equivalent amount of 30% sucrose solution was added to uninfected A549, which was used as a control. At 15 h post-infection (p.i.), total cellular RNAs were harvested by Trizol. The RNA pellets were then suspended in 10 mL of 5 × SSC buffer (diluted from 20 × SSC buffer, Corning Inc., Life Sciences, Acton, MA, USA). To purify tRNA-GluCTC, biotinylated DNA oligos (which are complementary to tRF5-GluCTC) were added to the RNA suspension. The mixture was then heated to 95 °C for 3 min and subsequently incubated at 65 °C for 15 min, followed by another incubation at room temperature for 3 h. The DNA-tRNA complex was then pulled down by adding streptavidin agarose beads (Thermo Scientific, Waltham, MA, USA), which were pre-washed twice in 5 × SSC buffer, followed by rotation at 4 °C overnight. The beads were washed once with 1 × SSC buffer and three times with 0.1 × SSC buffer before DNase I treatment to degrade biotinylated-DNA oligos. The released RNAs from the DNA-RNA complex were then extracted using Trizol LS reagents, followed by 15% denaturing polyacrylamide gel. The tRNA bands were cut and followed by PAGE gel elution using ZR small-RNATM PAGE Recovery Kit (Zymo Research, Orange, CA, USA). The purified tRNAs were then under acid hydrolysis degradation and lyophilization as previously described for RNA 2D-HELS MS sequencing [25,26,27].

### 2.6. tRNA-GluCTC Sequencing

tRNAs are enriched with chemical modifications, which are dynamic and sensitive to multiple post-transcriptional regulatory mechanisms. MS, in particular combined liquid chromatography and tandem MS spectrometry (LC-MS/MS), is recognized as the ‘gold standard’ for RNA modification analysis. However, it involves the full digestion of RNAs to component nucleotides and cannot provide full location or modification information. Recent works by Zhang et al. have developed a series of novel MS-based sequencing methods, referred to as MassSpec-Seq, that can simultaneously sequence RNA directly and quantify different RNA modifications with single-nucleotide stoichiometric precision through a complete set of MS ladders obtained from random RNA cuts and sequence mapping [25,26,28]. This method was further improved by an MS ladder complementation sequencing approach (MLC-Seq) [29]. By using MLC-Seq on tRNA-GluCTC obtained from human mock-infected or RSV-infected A549 cells, we tracked the location and stoichiometric changes of tRNA-GluCTC nucleotides caused by RSV infection. In brief, samples were analyzed using previously published protocols with minor modifications [29], and data were collected using an Orbitrap Exploris 240 mass spectrometer (ThermoFisher Scientific, Bremen, Germany) coupled to a Vanquish Horizon UHPLC. These experiments were performed using a DNAPac reversed phase (RP) column (2.1 mm × 50 mm, ThermoFisher Scientific) with 2% HFIP and 0.1% DIPEA as eluent A and methanol, 0.075% HFIP, and 0.0375% DIPEA as eluent B. A gradient of 20% to 80% B over 6.7 min was used for the analysis of intact RNA samples, while acid-degraded samples used a gradient from 15% to 35% B over 20 min.

### 2.7. NGS Sequencing of tRNA-GluCTC Sample

Pulled-down RNAs were delivered to the Genomics Core of UTMB for small RNA sequencing. RNAs were treated with T4 polynucleotide kinase (T4PNK) to convert the 3’ cyclic phosphate group into a hydroxyl group, as described in [12,15], before small RNA libraries were created using the New England Biolabs small RNA library protocol (New England Biolabs, Ipswich, MA, USA). Library construction used a two-step ligation process to create templates compatible with Illumina-based next-generation sequence (NGS) analysis. Where appropriate, RNA samples were quantified using a Qubit™ fluorometric assay (Thermo Fisher Scientific). RNA quality was assessed using a 2100 Bioanalyzer and the RNA 6000 Pico LabChip ^®^ (Agilent Technologies, Santa Clara, CA, USA). Library creation was conducted by sequentially adding a 3′ adapter sequence followed by a 5′ adapter sequence. A cDNA copy was then synthesized using ProtoScript^®^ II reverse transcriptase (New England Biolabs) and a primer complementary to a segment of the 3′ adapter. The template population was amplified in 15 cycles (94 °C for 30 s; 62 °C for 30 s; 70 °C for 30 s). The libraries were not size-selected, and all NGS libraries were indexed. The final concentration of all NGS libraries was determined using a Qubit™ fluorometric assay, and the DNA fragment size of each library was assessed using a DNA 1000 high-sensitivity chip and a 2100 Bioanalyzer (Agilent Technologies). Sequence analysis (2  ×  50 bases) was performed on an Illumina Hi-Seq 1500 using the TruSeq SBS v3 kit. Sequence counts of 10 or more after adaptor sequence removal were used for further classification, and small RNAs were mapped using Novoalign software as previously described [8].

### 2.8. Northern Blot (NB)

Northern hybridization for tRF/tRNA was performed as we previously described [8,30,31]. To summarize the process: RNAs, pulled down by antisense DNA oligos, were separated in 15% denaturing polyacrylamide gel with 7 mol/L urea and then transferred to a positively charged nylon membrane (Amersham Biosciences, NJ, USA). The membrane was hybridized with a ^32^P-labeled DNA probe inversely complementary to the tRF of interest in ULTRAhyb-Oligo solution (Life Technologies, NY, USA), followed by washing according to the manufacturer’s instructions.

### 2.9. RNA Interference

Transfections of siRNA into A549 cells were carried out at a final concentration of 100 nM, targeting ALKBH1 or scrambled negative control (Sigma, Woodland, TX, USA), using A549 transfection reagent Lipofectamine 2000 (ThermoFisher Scientific, Waltham, MA, USA) according to the manufacturer’s recommendations. After 24 h, A549 cells were mock infected or infected with RSV for 15 h at an MOI of 1.

### 2.10. Statistical Analysis

Experimental results were analyzed using Graphpad Prism 5 software. Patient group comparison was done by non-parametric statistics methods. Specifically, an unpaired two-tailed Mann-Whitney U test was used for the comparison of two independent groups. Single, double, and triple asterisks represent a *p*-value of 0.05, 0.01, and 0.001, respectively. Means ± standard errors (SE) are shown. Correlation analyses were performed using Spearman’s rank correlation test, and Spearman’s rank correlation coefficient (RS) was used to determine correlations. A *p*-value of less than 0.05 was considered significant. For the statistical analysis to determine the impact of ALKBH1 on RSV replication and tRF induction in cells, the statistical significance was determined using analysis of variance (ANOVA). A *p*-value of less than 0.05 was considered significant. Means ± SE are shown.

## 3. Results

### 3.1. tRF Expression in Nasopharyngeal Swab (NPS) Samples

**Figure 1:**
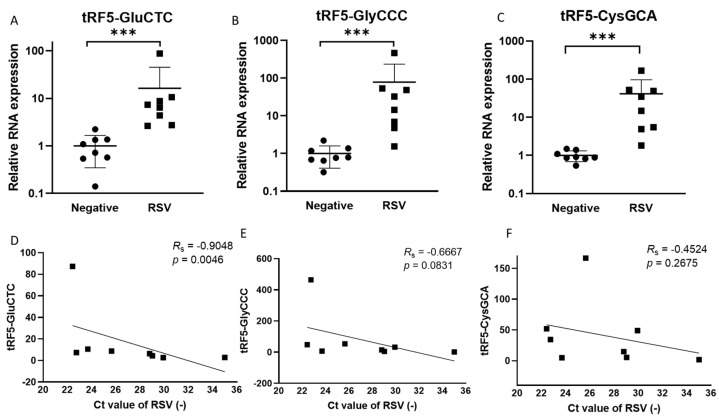
Changes in the expression of tRF5s in NPS samples by RSV. qRT-PCR was performed to detect (**A**) tRF5-GluCTC, (**B**) tRF5-GlyCCC, and (**C**) tRF5-CysGCA in the NPS from RSV and control (CN) patients. An unpaired two-tailed Mann-Whitney U test was used to compare two independent groups. The three asterisks represent a *p*-value of 0.001. For correlation analyses for tRF5-GluCTC (**D**), tRF5-GlyCCC (**E**), or tRF5-CysGCA (**F**) with NPS viral genome copies, we performed Spearman’s rank correlation test. Spearman’s rank correlation coefficient (R_S_) was used to determine correlations. A *p*-value of less than 0.05 was considered significant.

The induction of functional tRF5s from a limited set of parent tRNAs by RSV has been demonstrated in airway epithelial cells [8,11]. The present study compared the tRF abundance in NPS collected from RSV-infected or virus-negative children to investigate whether such an induction also occurs in the course of naturally acquired infections. Table 1 and Table 2 list the patients’ information and qRT-PCR primers to measure tRFs in NPSs. As shown in Figure 1A–C, the expression of tRF5-CysGCA, tRF5-GlyCCC, and Gly5-GluCTC in NPSs was significantly higher in RSV-positive NPS samples than in virus-negative ones, consistent with RSV-changed tRFs in infected cells [8]. Such an increase was not observed for tRF5-HisGTG. We also found that tRF5-GluCTC expression correlated to the genome copies of RSV (Figure 1D), which is consistent with the reported function of tRF5-GluCTC in promoting RSV replication [8,13]. These preliminary data suggest a possible detrimental role of tRF in RSV infection.

### 3.2. Modifications of tRNA-GluCTC

**Figure 2:**
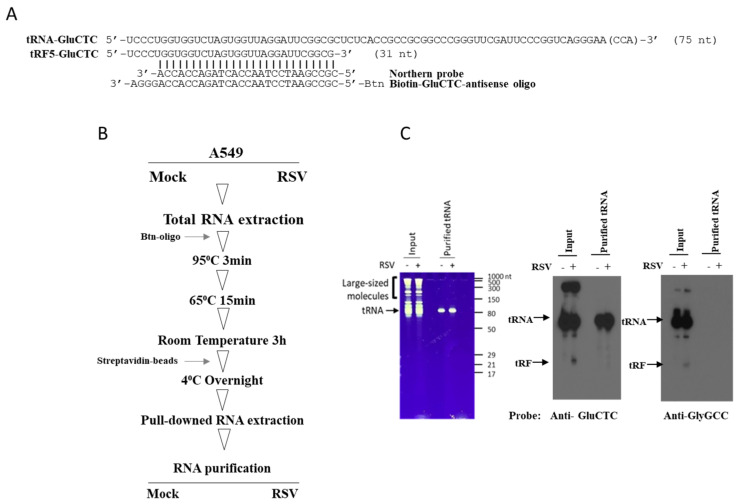
The schematic summaries on tRNA-GluCTC preparation. (**A**) Sequencing information on tRF5-GluCTC and its parent tRNA, the antisense probe used to detect tRNA by Northern blot, and biotinylated antisense oligo to pull down tRNA-GluCTC. (**B**) The workflow to purify tRNA-GluCTC. (**C**) The input and purified tRNA-GluCTC from mock- and RSV-infected cells. Left panel: SYBR^TM^ Green II RNA staining of total RNAs for mock- and RSV-infected cells (left two lanes) and enriched tRNAs (right two lanes). Middle panel: input RNAs and enriched tRNAs were detected by antisense oligo against tRNA-GluCTC. Right panel: input RNAs and enriched tRNAs were detected by antisense against tRNA-GlyGCC.

tRF induction by RSV is a tightly regulated process, as the infection only leads to the cleavage of one or very few isoforms among tRNA isoacceptors (tRNAs that have different anticodons but still carry the same amino acid) and isodecoders (tRNA genes that share the same anticodon but have different body sequences). tRNAs are enriched with chemical modifications, and their modification status plays a vital role in determining their interaction with ribonuclease and associated cleavage [32,33]. Therefore, the selective cleavage may result from the modification changes of parent tRNAs by RSV. Since tRF5-GluCTC is one of the most abundantly induced tRFs by RSV and also functionally important to RSV replication [8,13] and showed a correlation with viral genome copies in clinical samples (Figure 1D), the modifications of tRF5-GluCTC’s parent tRNA were investigated before and after RSV infection. To achieve a high resolution on modification identification, the parent tRNA of tRF5-GluCTC was enriched as shown in Figure 2. tRNA-GluCTC was incubated with biotinylated antisense oligo (Figure 2A) and subsequently pulled down by streptavidin beads, which was followed by gel separation and purification (Figure 2B). Figure 2C shows that tRNA-GluCTC from control and infected cells was successfully enriched, as the bands of purified samples can be detected by the probe against tRNA-GluCTC (middle panel) but not by the probe against tRNA-GlyGCC (right panel).

**Figure 3:**
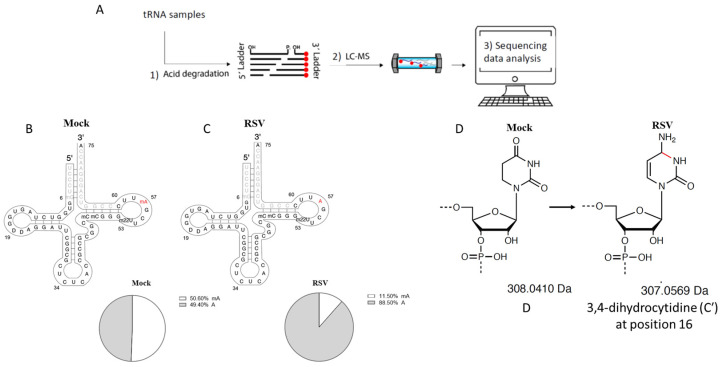
Identified tRNA modification changes by RSV. (**A**) Sequencing workflow. More detailed information can be found in Materials and Methods. (**B**,**C**) Stoichiometric changes of m1A at position 57 methylated nucleotides by RSV infection. The percentage of m1A at position 57 was reduced from 51% in the mock-infected cells (**B**) to 11% in RSV-infected cells (**C**). (**D**) The putative change of 1 Da on the pseudouritidine D by RSV.

The use of MLC-Seq on tRNA-GluCTC revealed a change in the methylation level of adenine at position 57 of the tRNA-GluCTC. About half of the tRNA-GluCTC stayed methylated in A57 for mock-infected cells (Figure 3B); this decreased to 11.5% after the infection (Figure 3C). The detailed measurement of methylation levels at position A57 in patient samples is provided in (Supplementary Excel). Additionally, a novel modification was discovered at positions 19 and 20 of tRNA-GluCTC. Both sites in mock-infected cells were identified to be 100% peudourantine (D). After infection, for the mass of 33% of D19 and 71% of D20 decreased by 1 Da. This was presumably caused by 3,4-dihydrocytidine (C′) at position 16, as described in Zhang et al. [29] (Figure 3D). Some methylation modifications were also observed on C and U. However, they were not changed by RSV infection.

### 3.3. Impact of Methylation Change on RSV Replication

**Figure 4:**
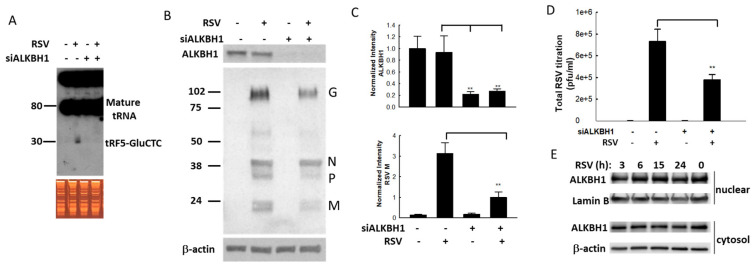
The impact of ALKBH1 on the RSV infection. (**A**) ALKBH1 gene knockdown prevents the induction of tRF5-GluCTC by RSV. The input of total RNAs in mock- and RSV-infected cells were checked by EB staining and induced tRF5-GluCTC was determined by Northern blot. (**B**,**C**) ALKBH1 gene knockdown decreased the expression of RSV proteins. The ALKBH1 gene knockdown was confirmed by Western blot using an anti-ALKBH1 antibody. The membrane was stripped and reprobed for RSV proteins and β-actin (**B**). Densitometric analysis of the band intensity of ALKBH1 was performed using the histogram function of Adobe Photoshop. Results are shown after corrections to β-actin. Results are representative of three independent experiments (**C**). (**D**) The impact of ALKBH1 on RSV virus titration was also determined. The double asterisk represents *p* values of 0.01. Data are shown as means ± SE. (**E**) The nuclear and cytosolic expression of ALKBH1 along RSV infection. The membranes were stripped and reprobed for lamin B as a loading control for nuclear fraction, while β-actin was used as a loading control for cytosol fraction.

The demethylation of A57 by RSV may play an important role in tRF5-GluCTC induction, as tRNA cleavage has been shown to be sensitive to this modification [34]. The change is thought to impact tRNA structure, stability, and function, which in turn can have a wide range of effects at the cellular level [35]. Of these modifications, m1A is common and has a major impact on tRNA stability [36]. AlkB Homolog 1 (ALKBH1) has been reported to be a tRNA demethylase by removing N(1)-methyladenine from various tRNAs, with a preference for N(1)-methyladenine at position 57/58 (m1A) present on a stem-loop structure of tRNAs. Therefore, ALKBH1-specific siRNAs were used first to suppress its expression and investigate its role in tRF5-GluCTC induction. In scrambled siRNA-treated cells, tRF5-GluCTC was detected in RSV-infected cells but not in mock-infected cells, indicating that the RSV infection induced tRF5-GluCTC (Figure 4A, first two lanes). This induction was suppressed by ALKBH1 knockdown (Figure 4A, last two lanes), suggesting the importance of ALKBH1 in tRNA-GluCTC cleavage. The effectiveness of ALKBH1-specific siRNA in silencing ALKBH1 expression was confirmed by Western blot using the antibody specifically against ALKBH1 (Cat#: ab126596, Abcam, Boston, MA) (Figure 4B,C). Suppression of ALKBH1 expression also led to decreased viral proteins (Figure 4B,C) and virus replication (Figure 4D). To investigate whether RSV uses ALKBH1 to control replication via controlling ALKBH1 expression, we measured ALKBH1 expression in the context of RSV infection in both cytosol and nuclear compartments. As shown in Figure 4E, the expression of ALKBH1 was not changed by RSV infection, suggesting that RSV-altered ALKBH1 activity affects the replication. This will be investigated in the future. These results are consistent with our previous findings showing the promotion of RSV replication by tRF5-GluCTC.

## 4. Discussion

tRFs are recently discovered sncRNAs and have quickly been found to be relevant to many diseases, including neurodegenerative diseases, cancers, and infectious diseases [7,9,15,37,38,39,40,41,42]. Since our first publication, which demonstrated that the most RSV-impacted sncRNAs in airway epithelial cells belong to tRFs [8], our team has investigated the functions of induced tRFs [8,11,12], identified the ribonucleases responsible for the tRNA cleavage [8,11,12], determined the platform through which tRFs carry out their gene regulatory functions [12], and outlined the flow work of tRF target identification [13]. The present study demonstrates the possible relevance of tRFs in RSV infection by showing tRF induction in NPS samples of infected patients and/or the direct correlation between expression levels of tRF5-GluCTC-CTC and RSV genome copies (Figure 1). In addition to tRF5-GluCTC, we also checked the expression of tRF5-GlyGCC-3, a tRF that is strongly induced by RSV in cells (~30 folds) [8]. We found that its expression levels also correlated with RSV replication, further supporting the clinical significance of tRFs in RSV infections (Supplementary Figure S1). This isoform of tRF5-GlyGCC-3 cannot be induced by SARS-CoV-2 and other viruses [9,43], suggesting virus-specific induction. In this study, no correlation between tRF5-CysGCA and RSV genome copies was found (Figure 1F), consistent with our previous finding showing that tRF5-CysGCA is not involved in regulating RSV replication. There was a trend showing the correlation between the expression of tRF5-GlyCCC and RSV genome (*p* = 0.08, Figure 1E), which may require more samples for confirmation. Nevertheless, the expression-viral genome correlation using limited sample numbers highlighted the possibility of using specific tRFs as biomarkers for viral infectious diseases.

In this study, we tried different methods to enrich tRNA-GluCTC as discussed in Drino et al. [44] and found the protocol summarized in Figure 2B reached a better result compared to the alternative Method 2 listed in Supplementary Figure S2. In brief, Method 2 used ion exchange chromatography followed by NaCl gradient elution as an essential step to enrich total tRNAs [44]. This step was found to be unnecessary and detrimental, however, as many tRNAs were wasted when 500 mM or greater NaCl was used to elute the larger RNAs (Supplementary Figure S2, right panels of B and C). In addition, the elongated processing may lead to potential RNA degradation. The method shown in Figure 2B and Method 1 in Supplementary Figure S2 were both found to enrich the desired tRNA; the method shown in Figure 2B was chosen because of its shorter exposure of RNAs to the annealing temperature of 60 °C.

As mentioned, tRNAs are enriched with chemical modifications. Such complexity, along with the existence of isoacceptor and isodecoder tRNAs, makes it hard to identify impacted tRNA modifications in response to environmental changes. Next-generation sequencing (NGS)-based RNA sequencing (RNA-Seq) has advanced the discovery of novel functional RNAs. However, it is limited in identifying modifications because the sequencing substrates are the reverse transcribed cDNA rather than the RNA itself. RNA-seq in combination with a pull-down using a modification-specific antibody is also limited in its ability to identify multiple tRNA modifications simultaneously. Nanopore-based RNA-Seq has been used recently for the sequencing of tRNAs [45], but it suffers from a high error rate and cannot reach a single-nucleotide resolution for RNA modifications. LC-MS/MS, as mentioned, cannot locate the modifications because it is restrained to the ribonucleoside level, losing information regarding the location and co-occurrence of modified nucleotides [46,47,48]. The MLC-Seq methods under continuing development by Shenglong Zhang’s lab, which combine MS analysis of RNA ladders obtained from randomly cut RNAs with complementary NSG mapping, are capable of sequencing RNA directly and simultaneously quantifying different RNA modifications with single-nucleotide stoichiometric precision [25,26,28,29]. In addition to identifying RSV-demethylated A^57^, this approach revealed a novel modification, i.e., a 1 Da reduction of D^19^ and D^20^; the associated modification mechanisms, regulatory enzymes, and the role of 1 Da reduction by RSV infections would need to be clarified in the future.

Overall, this study revealed a new mechanism controlling RSV replication. The importance of post-transcriptional modification of tRNAs in tRF biogenesis and the identification of the associated enzyme responsible for the modification provide critical insight that can lead to better strategies for antiviral developments to counter RSV infection.

## Figures and Tables

**Table 1:** Patient gender, age, and race information.

	Control (CN)	RSV Patient
No. of patients	8	9
Gender (Male:Female)	5:3	5:4
Mean age (months (range))	7.68 (0.75~14)	7.88 (1.25~16)
Race (African American:Caucasian:Asian)	2:5:1	3:6:0

**Table 2:** Primer information to detect tRF5s using qRT-PCR.

tRF5–GlyCCC	Sequence	GCAUUGGUGGUUCAGUGGUAGAAUUCUCGCC
	Forward primer	GCATGGGTGGTTCAGTG
	Reverse primer	CGTCGGACTGTAGAACTCTCAAAGC
tRF5–GluCTC	Sequence	UCCCUGGUGGUCUAGUGGUUAGGAUUCGGCGCU
	Forward primer	TCCCTGGTGGTCTAGTG
	Reverse primer	CGTCGGACTGTAGAACTCTCAAAGC
tRF5–CysGCA	Sequence	GGGUAUAGCUCAGUGGUAGAGCAUUUGACUGC
	Forward primer	AGTGGTAGAGCATTTGACTGC
	Reverse primer	CGTCGGACTGTAGAACTCTCAAAGC

## Data Availability

The data presented in this study are openly available in Viruses.

## Supplementary Materials

The following supporting information can be downloaded at: https://www.mdpi.com/article/10.3390/v15010057/s1, Figure S1: RSV infection affects the expression of tRF5-GlyGCC-3.; Figure S2: Additional tRNA purification methods.; Supplementary Excel.
