# Supplementary material for: Parent tRNA Modification Status Determines the Induction of Functional tRNA-Derived RNA by Respiratory Syncytial Virus Infection
Source: Viruses. 2022 Dec 24;15(1):57. doi: 10.3390/v15010057 (PMC9860972; doi:10.3390/v15010057)
Supplement: Supplementary file 1 [file viruses-15-00057-s001.zip › viruses-2031706-supplementary.pdf]

**tRNA-GlyGCC-3** 5′-GCAUUGGUGGUUCAGUGGUAGAAUUCUCG-3′

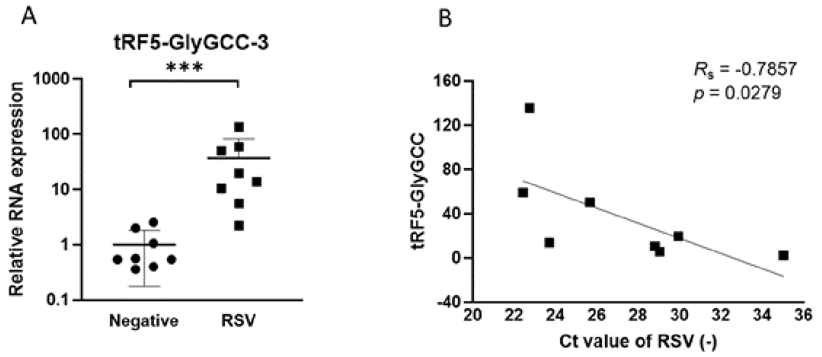


**Supplementary Figure S1** RSV infection affects the expression of tRF5-GlyGCC-3. (**A**) qRT-PCR was performed to detect tRF5-GlyGCC-3 in the NPS from RSV and CN patients. An unpaired two-tailed Mann-Whitney U test was used to compare two independent groups. Triple asterisks represents a $p$-value of <0.001. (B) The expression of tRF5-GlyGCC-3 correlated with RSV genome copies. We performed Spearman's rank correlation test. Spearman's rank correlation coefficient (Rs) was used to determine correlations. A $p$-value of less than 0.05 was considered significant.

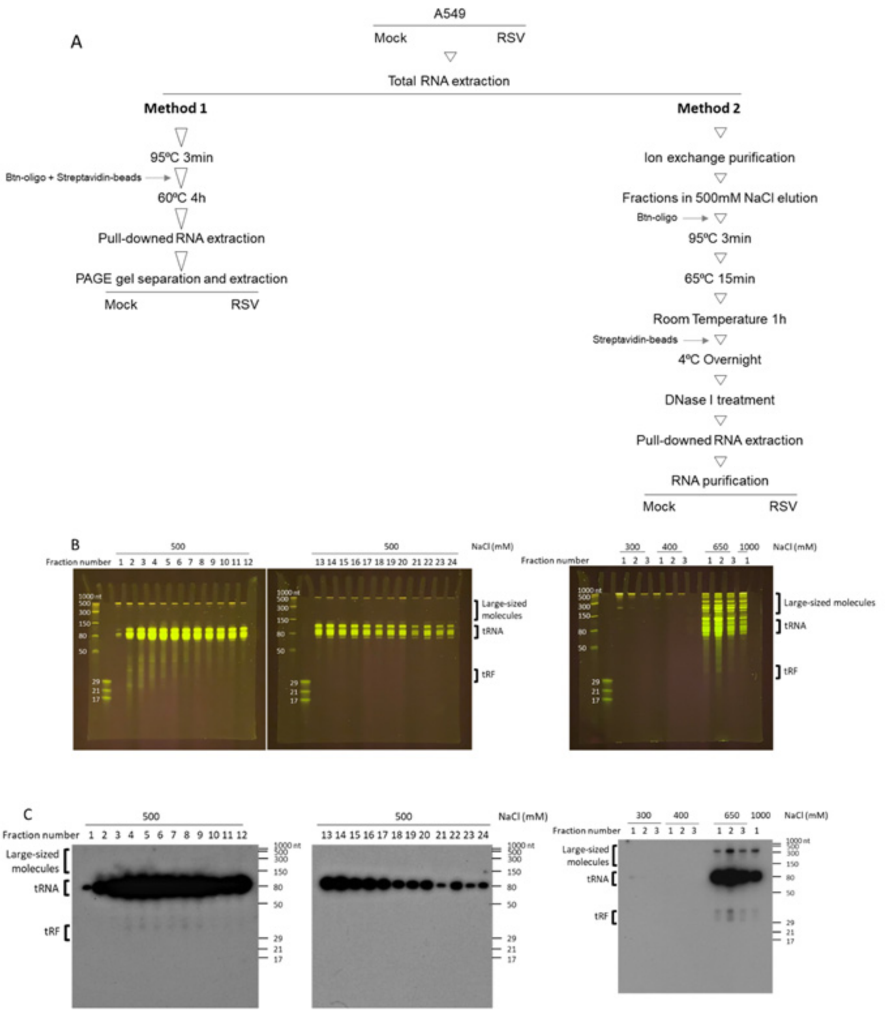


**Supplementary Figure S2** Additional tRNA purification methods. (**A**) Two alternate workflows to purify tRNA -GluCTC. (**B-C**). The RNAs loaded in ion exchange column were eluted with different concentrations of NaCl. The eluted RNA fractions were loaded to the 15% denaturing polyacrylamide gel with 7 mol/l urea, followed by SYBRTM Green II RNA staining (**B**) or transferring to a positively charged nylon membrane for Northern blot using a $^{32}$P-labeled DNA probe reversely complementary to the tRNA of interest in ULTRAhyb-Oligo solution (**C**).
